# Fetuin-A: a relevant novel serum biomarker for non-invasive diagnosis of metabolic dysfunction-associated steatotic liver disease (MASLD): a retrospective case-control study

**DOI:** 10.1186/s12876-024-03310-y

**Published:** 2024-07-18

**Authors:** Mohamed M. Elhoseeny, Badawy A. Abdulaziz, Mohamed A. Mohamed, Radwa M. Elsharaby, Ghadeer M. Rashad, Amira A. A. Othman

**Affiliations:** 1 Department of Internal Medicine, Faculty of Medicine, Suez University, Suez, Egypt; 2 Department of Hepatology, Gastroenterology, and Infectious Diseases, Faculty of Medicine, Benha University, Benha, Egypt; 3 Department of Clinical Pathology, Faculty of Medicine, Tanta University, Tanta, Egypt

**Keywords:** CAP scan, Fetuin-A, Fibroscan, NAFLD

## Abstract

**Objectives:**

To determine how fetuin-A contributes to diagnosing and assessing MASLD severity.

**Methods:**

Fifty MASLD patients and fifty healthy control participants were involved in this retrospective case-control research. Abdominal ultrasonography, fibroscan with controlled attenuated parameter scan (CAP scan), laboratory investigation (including fetuin-A assessment), clinical examination, and history-taking were performed on every case.

**Results:**

Fetuin-A level was considerably higher in the Cases group (1154.85 ± 629.89) than in the Control group (505.29 ± 150.4) (*p* < 0.001). Fetuin-A had significant validity in the prediction of MASLD at a cut-off > 702.5 with 82% sensitivity, 90% specificity, and 86% overall accuracy.

**Conclusion:**

One possible marker for MASLD diagnosis could be fetuin-A. Furthermore, a substantial association between such marker and the severity of the disease as it revealed a significant correlation with ultrasound grading and fibroscan with controlled attenuated parameters.

**Trial registration**

***1- Pan African Clinical Trial Registry.***

Unique Identifying number/registration ID: PACTR202309644280965.

URL: https://pactr.samrc.ac.za/TrialDisplay.aspx?TrialID=26860.

Registration Approval date: 21/09/2023.

***2- ClinicalTrials.gov***.

Unique Identifying number /registration ID: NCT06097039.

URL: https://clinicaltrials.gov/study/NCT06097039?cond=NCT06097039&rank=1.

Registration Approval date: 25/10/2023.

## Highlights

Metabolic Dysfunction-Associated Steatotic Liver Disease (MASLD) is rapidly becoming one of the most common causes of chronic liver disease worldwide.

Liver biopsy is the gold standard tool for chronic liver disease diagnosis, but it is usually not accepted by patients due to its invasive nature.

Fetuin-A could be a promising non-invasive marker for the diagnosis of MASLD with a sensitivity of 82%, specificity of 90%, and accuracy of 86%.

## Background

“Non-alcoholic fatty liver disease (NAFLD)” is a broad expression that encompasses a variety of conditions, including “non-alcoholic fatty liver (NAFLD, steatosis)”, intrahepatic lipid accumulation without any secondary cause of liver fat accumulation such as heavy alcohol consumption, of at least 5% of the liver weight that causes liver dysfunction and inflammation, and advance to “non-alcoholic steatohepatitis (NASH)”, which is distinguished by liver fibrosis, which advances to cirrhosis, and hepatocellular carcinoma in about 30–40% of cases [1]. The principal limitations of the terms NAFLD and NASH are the reliance on exclusionary confounder terms and the use of potentially stigmatizing language. The name chosen to replace NAFLD was metabolic dysfunction-associated steatotic liver disease (MASLD) [2]. MASLD is a serious challenge because of its prevalence, difficult diagnosis, complex etiology, and lack of recognized treatments [3]. It can be a consequence in non-drinker obese or diabetic patients (primary), affecting about 30–40% of the adult population and up to 95% of obese people. It may be a consequence of a toxin or drug (secondary) [4].

Imaging, including “ultrasonography (US)”, “computed tomography (CT)”, and “magnetic resonance imaging (MRI)”, are often used to diagnose MASLD incidentally when routine liver function lab shows abnormal biochemical test results. The controlled attenuation parameter (CAP) represents a US-based quantitative, and non-invasive diagnostic tool for MASLD [5]. A liver biopsy, required to determine the fibrosis advancement, is intrusive and expensive [4], that is why extensive researches have been undertaken to identify relevant blood biomarkers for the early prediction of NAFLD.

Fetuin-A is a plasma carrier glycoprotein that is primarily produced by hepatocytes and is released into the bloodstream to aid in the transportation and availability of a wide range of drugs. It was originally known as phosphoprotein 63 KDa (pp63) or countertrypin. Its potential application as a biomarker for the diagnosis of MASLD has been studied [6]. It is expressed most frequently in adult hepatocytes and embryonic cells, and less frequently in monocytes and adipocytes. Fetuin-A has a wide range of physiological and pathological roles and binds to a variety of receptors. It plays a role in controlling the insulin signaling system, osteogenesis, and calcium metabolism. In addition to these multiple roles, it also plays the roles of an ectopic calcification inhibitor, protease inhibitor, inflammatory mediator, anti-inflammatory partner, atherogenic factor, and adipogenic factor [7]. Additionally, it has been shown that Fetuin-A plays a critical role in the pathophysiology of a number of diseases, including metabolic disorders, nonalcoholic fatty liver disease (NAFLD), insulin resistance (IR), type 2 diabetic mellitus (T2DM), cancers, and brain problems [8].

Our objective is to ascertain the extent to which relevant fetuin-A aids in the MASLD diagnosis and severity evaluation.

## Methods

### Study design and setting

Retrospective case-control research conducted at Benha University’s hepatology, gastrointestinal, and infectious diseases departments, Egypt. This investigation was planned to be conducted between December 2021 and December 2022. **The study was conducted after being approved by the Benha University local ethics commission. Approval #: 1.8.19201. All contributors gave their voluntary, written, informed consent for the release of any connected photographs to meet the STROCSS standards** [9].

### Subjects

This study included 100 individuals who were divided into two groups: **Group I** “Cases group” consisted of 50 MASLD patients diagnosed using liver biochemistry, abdominal ultrasound, and Fibroscan with CAP, and **Group II** “Control group” consisted of 50 healthy subjects who had normal liver function as determined by transabdominal ultrasonography and normal liver enzyme levels.

Patients with liver cirrhosis were included, they represented ascites, jaundice, enlarged spleen, exhaustion, red palms, and other complications.

Pregnant women, patients under 18 years of age, chronic heavy alcohol drinkers “more than 40 grams a day for men and 20 grams a day for women”, patients with hepatobiliary cancers, patients with viral hepatitis like HBV or HCV, patients with chronic autoimmune hepatitis, Wilson disease, or hemochromatosis were rolled out from the study. The study also excluded participants who used steatogenic drugs like Amiodarone, Valproic Acid, and Tetracycline, antiretroviral medications, disease-modifying anti-rheumatic medication like methotrexate, or medications used to treat MASLD like vitamin E, and thiazolidinediones. Patients receiving metformin, glucagon-like peptide-1 (GLP-1) agonists, or sodium-glucose cotransporter-2 (SGLT2) Inhibitors were also excluded.

### Clinical and biochemical characterization

#### Clinical examination

Each patient’s clinical and pathological data were recorded including a full medical history and a detailed clinical examination, as well as symptoms of chronic fatigue, hypertension, and chronic liver disease.

#### Anthropometric evaluation

Body mass index “BMI” was calculated from measurements of height and weight. BMI has been graded accordingly into under-weight, optimum-weight, over-weight, and obese “< 18.5, < 24.9, < 25.0–29.9, and < 30.0 respectively” [10].

#### Lab biochemical assay

- Quantitative measurement of human fetuin-A, in serum, using sandwich enzyme-linked immunosorbent assay “Sandwich-ELISA kit: Epitope Diagnostics Inc.™, CatLog no. KT 800” following the manufacturer operating guidelines.
- MASLD Fibrosis score calculation formula [11]:
  “-1.675 + 0.037 × age (years) + 0.094 × BMI (kg/m2) + 1.13 × IFG/diabetes (yes = 1, no = 0) + 0.99 × AST/ALT ratio – 0.013 × platelet (×109/l) – 0.66 × albumin (g/dl)”.

### Imaging tests

- *Abdominal ultrasonography*:

A “real-time gray-scale device” with 3.5 MHZ frequency was utilized in ultrasonography patients’ examination using. Patients were examined while fasting for 6 h and took anti-flatulent to get rid of gases. Scanning was done while patients were in a supine position.

Steatosis is often classified employing a particular scoring system; “Absent (S0): normal liver echogenicity. Mild (S1): a slight and diffuse increase in liver echogenicity with normal periportal and diaphragmatic representation; moderate (S2): a moderate, diffuse increase in liver echogenicity with impaired portal vein wall and the diaphragm representation; Severe (S3): a marked diffuse increase in liver echogenicity with obscuring portal vein wall and the diaphragm representation” [12].

- *Fibroscan with Controlled Attenuated Parameter (CAP measurement)*:

The CAP is a novel technique added to the FibroScan^®^ device for the non-invasive steatosis scoring assessment.

The “FibroScan^®^ 502 device, Echosens, Paris, France” equipped with either the 3.5 MHz M-probe or 2.5 MHz XL-probe developed recently for obese subjects, is used to assess CAP and liver stiffness measurement (LSM). The value is expressed in decibels per meter (dB/m), ranging from 100 to 400 dB/m, and kilopascal (kPa) ranging from 2.5 to 75 kPa. A median CAP of at least 236 dB/m indicates liver steatosis. A reliable LSM measurement is obtained when having at least 10 valid shots and a success rate of ≥ 60% and an interquartile range (IQR) < 30% of the median LSM value [13]. In liver biopsies, three grades of steatosis can be determined according to the level of CAP: “absent (S0 < 236 dB), mild (S1 ≥ 236 dB), moderate (S2 ≥ 270), and severe (S3 ≥ 302 dB)” [13]. The optimal cut-off LSM values to define advanced fibrosis (≥ F3) and cirrhosis (F4), varied considerably according to the probe used: “F3 = 9.6–11.4 kPa, F4 ≥ 11.5 kPa” using M-probe, and “F3 = 9.3–10.9 kPa, F4 ≥ 11.0 kPa” using XL-probe [14].

### Statistical analysis

“SPSS^®^” program (IBM, SPSS Inc, USA) was used to code, process, and analyze the data. “Median ± SD” was used to display the data. “Numbers (frequency)” and “percentages” representing qualitative data were displayed. “The chi-square test” was used to compare the groups. “The Kolmogorov-Smirnov test” examined the normality of quantitative data. If the data were abnormally distributed, then “the Kruskal Wallis test” was applied; otherwise, “one-way ANOVA test” was used to compare normally distributed quantitative variables of the groups. “The receiver operator characteristic curve (ROC)” is used to define “the true-positive rate” (Sensitivity) as a function of “the false-positive rate” (Specificity). *P* < 0.05 is considered significant for all tests.

## Results

There were no statistically significant variations in age, or sex, among the studied groups, indicating that they were homogeneous. In terms of BMI, the studied groups differed statistically significantly. In the Cases group, 58% of the patients had diabetes mellitus, 42% had hypertension, and 98% had hyperlipidemia (Table 1).

**Table 1:** Demographic data of the studied groups

Parameter		Cases group *N* = 50	Control group *N* = 50	*P*
Sex	Female	25(50%)	26(52%)	0.08
	Male	25(50%)	24(48%)	
Age (years)		42.46 ± 10.41	40.62 ± 11.63	0.06
BMI (kg/m2)		46.49 ± 7.02	32.22 ± 10.16	0.61
Diabetes mellitus		29(58%)	0	
Hypertension		21(42%)	0	
Hyperlipidemia		49(98%)	0	

The mean LSM score was 6.58 with a range of 3.1 to 24.1 and the most frequent class in LSM fibroscan was F0 & F1 (38% & 36% respectively). The CAP score among the studied cases ranged from 222 to 372 with a mean of 287.5. The most frequent CAP class was S 3 (52%). The most frequent US grade among the studied cases was 3 (52%) (Table 2).

**Table 2:** Imaging findings among studied groups

Parameter		Cases group *N* = 50	Control group *N* = 50
LSM fibroscan	F 0	19 (38%)	
	F 1	18 (36%)	
	F 2	4 (8%)	
	F 2–3	3 (6%)	
	F 3 F 4	4 (8%) 2 (4%)	
	Mean ± Sd	6.58 ± 3.35	
	Median (Range)	5.7 (3.1–24.1)	
CAP score	S 1	9 (18%)	
	S 2	15 (30%)	
	S 3	26 (52%)	
	Mean ± Sd	287.5 ± 44.62	
	Range	222–372	
US grade	1	9 (18%)	
	2	15 (30%)	
	3	26 (52%)	

The cases group had a statistically significant higher platelet count, FBS, and 2-hour postprandial blood sugar than the control group. When compared to the control group, cases showed a statistically significant increase in cholesterol, TG, and LDL, as well as a statistically significant drop in HDL. In addition, the cases group had a statistically significant higher total bilirubin level than the control group. There was a statistically significant increase in cases group fetuin-A when compared to the control group (Table 3).

**Table 3:** Laboratory findings among the studied groups

Parameter		Cases group *N* = 50	Control group *N* = 50	*P*
Hb (gm/dl)	Mean ± Sd	13.54 ± 0.98	13.43 ± 1.01	0.60
	Range	11.5–15.2	11.5–15	
WBC (x10^3^/mm3)	Mean ± Sd	4.72 ± 1.17	4.98 ± 1.63	0.36
	Range	3–8	2.5–8.5	
Platelets (x10^3^/mm3)	Mean ± Sd	269.86 ± 68.76	240.08 ± 50.85	0.02
	Range	160–400	150–350	
FBS (mmol/l)	Mean ± Sd	117.4 ± 27	82.86 ± 7.77	< 0001
	Range	75–170	75–120	
2 h Post prandial (mmol/l)	Mean ± Sd	193.42 ± 67.81	110.24 ± 14.03	< 0.001
	Range	90–320	90–160	
Cholesterol (mg/dl)	Mean ± Sd	193.8 ± 26.53	164.2 ± 17.25	< 0.001
	Range	150–250	124–195	
Triglyceride (mg/dl)	Mean ± Sd	176.86 ± 15.63	135.9 ± 17.51	< 0.001
	Range	140–220	100–180	
LDL (mg/dl)	Mean ± Sd	100.57 ± 14.28	81.04 ± 14.61	< 0.001
	Range	75–140	40–100	
HDL (mg/dl)	Mean ± Sd	44.82 ± 12.84	65.36 ± 7.92	< 0.001
	Range	24–85	45–80	
ALT (U/L)	Mean ± Sd	19.06 ± 8.6	18.28 ± 6.74	
	Median	16	18	0.92
	Range	8–42	10–34	
AST (U/L)	Mean ± Sd	19.4 ± 7.84	18.5 ± 5.79	
	Median	12	18	0.48
	Range	7–38	10–35	
T. Bilirubin (mg/dl)	Mean ± Sd	0.73 ± 0.18	0.66 ± 0.18	0.04
	Range	0.4-1	0.45-1	
D. Bilirubin (mg/dl)	Mean ± Sd	0.36 ± 0.12	0.34 ± 0.11	0.29
	Range	0.1–0.6	0.2–0.6	
Albumin (gm/dl)	Mean ± Sd	4.09 ± 0.47	4.22 ± 0.56	0.20
	Range	3.2-5	3-5.1	
Fetuin-A (ng/ml)	Mean ± Sd Median	1154.85 ± 629.89 879	505.29 ± 150.4 496	< 0.001
	Range	592–2400	150–788	
MASLD F score	Mean ± Sd	-2.10 ± 1.66	-------	------
	Median	-20.8		
	Range	-4.5 / 2.88		

Fetuin-A levels were statistically significantly higher in S2 and S3 cases than in S1, as well as in US grade 3 cases than in grades 1 and 2 (Table 4).

**Table 4:** Relation between Fetuin-A and LSM, CAP score, and US grade among the studied cases group

Parameter		*N*	Fetuin-A		*P*
			Mean ± SD	Median	
LSM	F0	19	1210.13 ± 662.62	893	0.26
	F1	18	1045.73 ± 554.3	829.8	
	F2	4	1357.38 ± 794.39	1206.25	
	F2-3	3	1873.4 ± 870.16	2351.7	
	F3	4	884 ± 221.42	891	
	F4	2	670.45 ± 110.95	670.45	
CAP score	S1	9	808.96 ± 623.27	806	0.03
	S2	15	1232.89 ± 669.74	896	
	S3	26	1271.13 ± 635.3	903	
US grade:	1	9	1041.36 ± 522.21	806	0.04
	2	15	1085.22 ± 630.29	858	
	3	26	1343.63 ± 684.08	917	

Among the cases group, there was a statistically significant positive connection between Fetuin-A and both the CAP and MASLD scores. There was no statistically significant relationship between Fetuin-A and any of the examined parameters in the control group (Table 5).

**Table 5:** Correlation between Fetuin-A and different parameters among the studied cases and control groups

Parameter	Fetuin-A [Cases group] (*n* = 50)		Parameter	Fetuin-A [Control group] (*n* = 50)	
	*r*	*P*		*r*	*P*
Age	-0.05	0.74	Age	0.10	0.51
BMI	0.25	0.08	BMI	0.11	0.44
LSM fibroscan	0.07	0.65	Hb	0.03	0.82
CAP score	0.34	0.02	WBC	0.09	0.52
MASLD score	0.49	< 0.001	Platelets	0.08	0.59
Hb	0.03	0.81	FBS	0.06	0.71
WBC	0.14	0.35	Post prandial	0.13	0.38
Platelets	0.11	0.44	Cholesterol	0.13	0.35
FBS	0.09	0.55	Triglyceride	0.09	0.52
Post prandial	0.03	0.81	LDL	0.22	0.12
Cholesterol	0.27	0.06	HDL	-0.02	0.88
Triglyceride	0.11	0.44	ALT	0.06	0.66
LDL	0.08	0.60	AST	0.04	0.76
HDL	-0.21	0.14	T. Bilirubin	0.09	0.53
ALT	0.10	0.48	D. Bilirubin	0.03	0.86
AST	0.08	0.59	Albumin	-0.06	0.70
T. Bilirubin	0.07	0.65			
D. Bilirubin	0.11	0.45			
Albumin	0.05	0.71			

Fetuin-A had a significant validity in the prediction of the non-alcoholic fatty liver at cut-off > 702.89 with a sensitivity of 82%, specificity of 90%, and accuracy of 86% (Table 6).

**Table 6:** Diagnostic performance of Fetuin-A in the prediction of non-alcoholic fatty liver among the studied groups

Cut off	AUC (95%CI)	Sensitivity	Specificity	PPV	NPV	Accuracy	*P*
> 702.891	0.95 (0.91–0.99)	82%	90%	89.1%	83.3%	86%	< 0.001

*AUC* Area under curve, *CI* Confidence interval, *PPV* Positive predicted value, *NPV* Negative predicted value

**Fig. 1:**
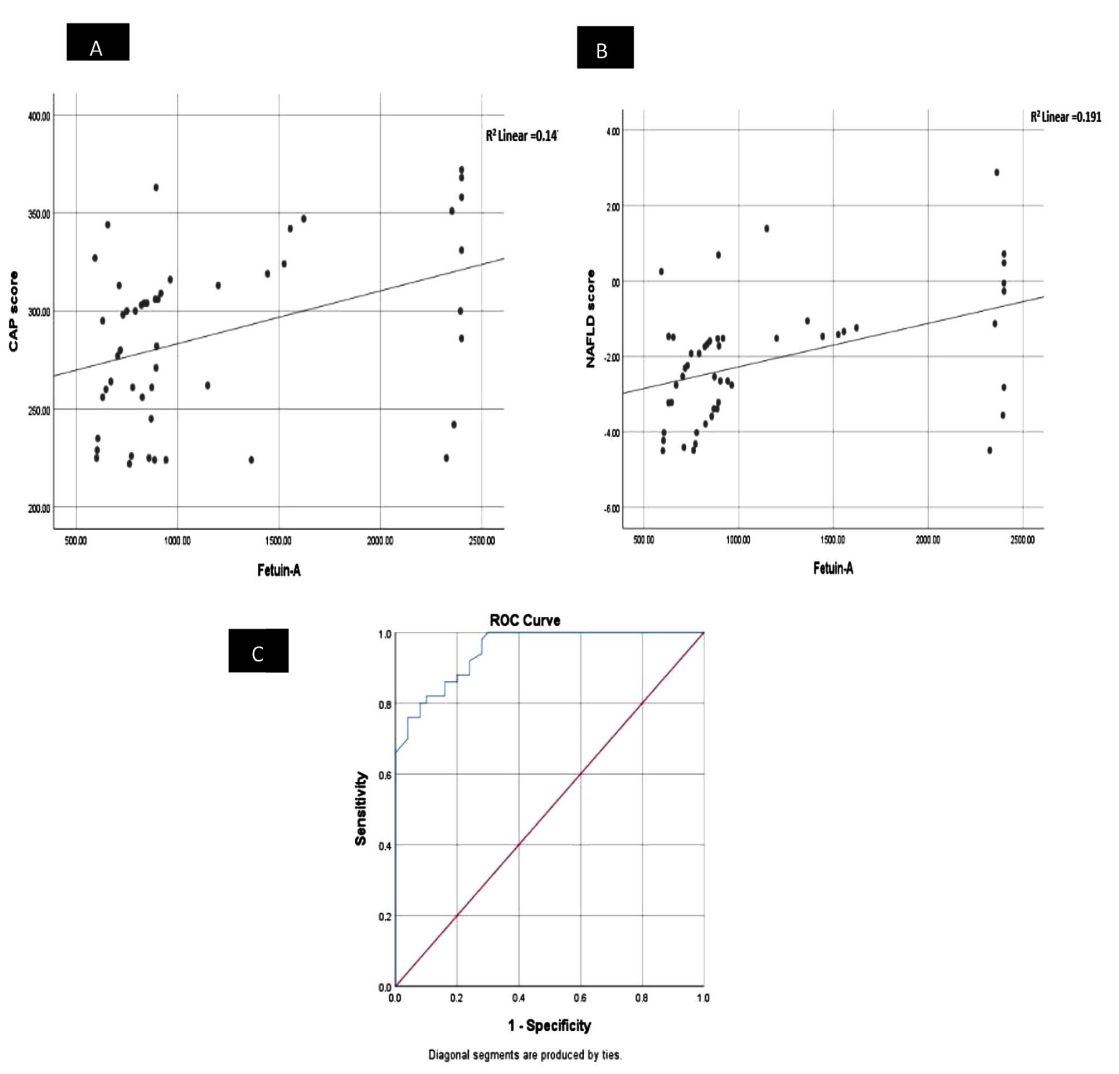
(**A**): Correlation between Fetuin-A and CAP score among the studied cases group, (**B**): Correlation between Fetuin-A and MASLD score among the studied cases group, (**C**): Roc curve analysis for Fetuin-A in the prediction of non-alcoholic fatty liver among the studied groups

The study found a correlation between Fetuin-A and CAP score among the studied cases (Fig. 1, A), a correlation between Fetuin-A and MASLD score among the studied cases (Fig. 1, B), and the Roc curve analysis showed the role of Fetuin-A in the prediction of MASLD among the studied groups (Fig. 1, C).

## Discussion

MASLD is diagnosed using three criteria: the patient must not be an alcoholic, steatosis must be detected using imaging or histology, and other liver disorders must be ruled out. “Liver biopsy” remains the most reliable way of diagnosing NASH. However, there are several downsides to liver biopsy [15]. Non-invasive, blood-based biomarkers with prognostic value in studies of MASLD patients: NAFLD fibrosis score (NFS), Fibrosis-4 (FIB-4), aspartate aminotransferase (AST) to platelet ratio index (APRI), enhanced liver fibrosis (ELF™), BARD (BMI, AST/ALT (alanine aminotransferase) ratio, diabetes), Hepamet Fibrosis Score (HFS), liver enzymes (AST + ALT), alpha-fetoprotein, platelet count, neutrophil to lymphocyte ratio (NLR), Lysyl oxidase-like (LOXL) 2, miR-122, liver stiffness, MEFIB (liver stiffness measured with magnetic resonance elastography (MRE) + FIB-4), and PNPLA3 GG genotype [16, 17].

Elevated fetuin-A levels have been associated with the development of a variety of liver-related metabolic issues, including IR, adipocyte inflammation, hepatocyte fibrosis, dyslipidemia, progressive macrophage infiltration, and increased toll-like receptor-4 (TLR4) expression resulting in obesity [18]. Recent clinical studies have found that a confirmed MASLD biopsy is related to elevated fetuin-A serum levels in conjunction with several MetS including poor glucose and lipid metabolism [19, 20]. Such findings unambiguously demonstrate that elevated fetuin-A levels in MASLD patients may be a helpful blood biomarker for early diagnosis [21, 22].

The current investigation found a statistically significant difference in BMI between cases and control groups, with mean values of 46.49 and 32.22 kg/m2, respectively. The rise in obesity, MetS, and T2D prevalence coincides with an increase in the incidence of NAFLD. Substantial prospective epidemiological studies have found a strong link between type 2 diabetes (T2D), obesity, and NAFLD. The three disorders are routinely treated as co-morbidities in various biomedical research settings. Patients with MASLD and NASH exhibited overall obesity rates of 51% and 81%, respectively with 60 to 95% prevalence [23].

Our study found that MASLD cases had a significantly higher platelet count than controls (269.86 vs. 240.08, respectively, but both groups were within normal ranges). Previous research supported our findings, as platelet count increased significantly in conjunction with MASLD compared to controls (251.36 vs. 246.22 respectively) [24, 25].

The elevated platelet count could indicate an inflammatory condition linked with MASLD [18, 19]. Platelets play a function in liver regeneration following liver damage, but they can also contribute to the development of liver fibrosis [24]. MASLD is characterized by “subclinical inflammation, and white blood cells (WBCs) and platelets” are frequent blood components used to detect systemic inflammation in such cases. Recently, “mean platelet volume (MPV), lymphocyte-monocyte ratio.

(LMR), platelet-to-lymphocyte count ratio (PLR), and neutrophil-to-lymphocyte ratio (NLR)” have all been postulated as potential novel biomarkers of the inflammatory process. It is still unclear whether inflammatory markers and MASLD are connected although numerous studies have found an association between MASLD and WBC count, MPV, and PLR [26].

In the present study, comparing cases to the control group, there was a significant rise of serum cholesterol (193.8 vs. 164.2 mg/dl respectively), serum TG (176.86 vs. 135.9 mg/dl respectively), serum LDL (100.57 vs. 81.04 mg/dl respectively). In addition to a significant decline of serum HDL (44.82 vs. 65.36 mg/dl respectively).

Dyslipidemia is characterized by “abnormally raised cholesterol, TG, and low-density lipoprotein cholesterol (LDL-C), as well as abnormally declined high-density lipoprotein cholesterol (HDL-C)” [27]. About 20–80% of MASLD patients have “atherogenic dyslipidemia, characteristically seen in patients with obesity, MetS, IR, and T2D, which is distinguished by increased blood levels of TG, LDL-C, and decreased blood levels of HDL-C” [28]. Extra fat builds up in hepatocytes as lipid droplets covered with a variety of structural proteins that may have a role in the etiology of liver disorders. Intrahepatic lipid buildup in MASLD is caused by anomalies in lipid metabolism, including reduced triglyceride (TG) export, increased liver uptake of free fatty acids, increased whole-body lipolysis, and increased synthesis of very low-density lipoproteins. These changes in lipid metabolism are associated with aberrant synthesis of adipokines (as adiponectin, resistin, and leptin) that impact signaling pathways, causing inflammatory - oxidative stress [29–31].

In this study, comparing cases vs. control group, there was a substantial rise in serum ALT (19.06 vs. 18.28 u/l) and serum AST (19.4 vs. 18.5 u/l) respectively.

Patients with MASLD may have “mild or moderate elevations in serum transaminases (ALT and AST), although normal aminotransferase levels do not rule out NAFLD” [32]. A high permeability of.

the hepatocyte cell membrane permits transaminases to seep into the bloodstream, as seen by higher levels of these enzymes, which are very vulnerable to liver injury. While AST elevation could be implicated in cardiac, hepatic, and skeletal muscle injuries, ALT is liver connected. Because of this, serum transaminases have been used as substitute markers for NAFLD. Higher hepatic fat fraction and intraabdominal visceral adipose tissue have been associated with higher blood transaminase concentrations [33]. As a result, individuals with “increased serum transaminases and fatty liver” have more severe “steatosis and visceral obesity” because of “insulin resistance-related metabolic abnormalities”. When insulin resistance first appears, affected individuals have normal serum transaminases and mild steatosis. Patients with advanced insulin resistance show substantial steatosis, elevated blood transaminases, and visceral adiposity [34].

In the present study, comparing cases to the control group, there was a significant increase of serum fetuin-A (1154.85 vs. 505.29 respectively).

Although a wide range of laboratory testing, imaging studies, and combinations of clinical and blood test data are nowadays available as non-invasive diagnostic markers for NAFLD, they are not sensitive enough or specific enough to differentiate between NAFL and NASH or to identify the progress of fibrosis [35]. Unfortunately, “liver biopsy” is still considered the “golden standard” for MASLD diagnosis and assessment of fibrosis progression [36]. Fetuin-A represents one of the newly adopted serum biomarkers for MASLD diagnosis of severity assessment [37]. Fetuin-A has been incriminated in the causation of several metabolic disorders for instance, dyslipidemia and hepatic steatosis [38–40]. Furthermore, it has been discovered that obesity [41], hypertriglyceridemia [42], T2D [43], and IR [44] are linked to elevated fetuin-A levels. As a result, the data presented above show that fetuin-A may be employed as a unique biological marker for the diagnosis of several metabolic illnesses including dyslipidemia, poor glycemic control, which all share responsibility for mitochondrial dysfunction, which increases reactive oxygen species (ROS) and eventually leads to MASLD [45].

In our investigation, we employed a cutoff value of 702.5 for serum fetuin-A, which resulted in 82% and 90% sensitivity and specificity, respectively, for identifying MASLD cases, with 86% diagnostic accuracy.

It is expected to find differences among different studies regarding the diagnostic ability of fetuin-A in MASLD cases diagnosis. These differences could be attributed to different sample sizes and methods of fetuin-A assessment in different ethical populations. The best “cutoff value” to distinguish between the MASLD group and control group regarding fetuin-A level was found to be ” > 500 with a sensitivity of 96.67%, a specificity of 100.0%, and an area under the curve (AUC) of 97.7%” in a prior similar study [46].

In our study, there was a significant positive correlation between serum fetuin-A and both CAP and MASLD scores, indicating the value of serum fetuin-A not only in the diagnosis but also in the assessment of the severity of MASLD cases. The more increased fetuin-A levels, the more hepatic steatosis is expected.

fetuin-A is produced by steatotic hepatocytes at early timepoints in MAFLD and correlates with insulin resistance both in mice and humans. In NASH, fetuin-A also co-localizes with activated liver macrophages and could be interpreted as a signal released by damaged hepatocytes [47]. Both preclinical and clinical research have demonstrated that elevated fetuin-A level is an indicator for several metabolic illnesses, such as obesity, T2D, NAFLD, NASH, IR, etc. [48, 49], which in turn cause several hepatic-related complications to progress to cirrhosis [50]. Fetuin-A modulation is associated with a number of pathophysiological variables that cause liver problems, including insulin receptor signaling deficiencies, adipocyte dysfunction, hepatic inflammation, fibrosis, triacylglycerol production, macrophage invasion, and TLR4 activation [35]. Furthermore, fetuin-A is essential for the pathophysiology of several inflammatory and metabolic diseases. It has recently been demonstrated that elevated fetuin-A levels induce mice’s adipocytes and macrophages to produce more inflammatory cytokines, which accelerates the development of many liver-related problems [48]. Hepatic sensitivity to high doses of pro-inflammatory cytokines leads to the histopathological changes seen in NASH [51]. This shows that hepatic cytokines play an important role in the transition from steatosis to NASH. One strategy to halt the progression of MASLD is to lower blood fetuin-A levels, which can be accomplished by treating a variety of conditions [52].

In the current study, serum fetuin-A did not have any significant correlation with any of the collected numerical variables in MASLD cases, apart from the two scores (MASLD and CAP score). Fetuin-A could be a promising non-invasive marker for the diagnosis of MASLD with a sensitivity of 82%, specificity of 90%, and accuracy of 86%.

Our study has some limitations. First of all, it included a relatively small sample size. Also, the impact of serum fetuin-A on patient prognosis should have been assessed. Finally, the effect of nutritional status on serum fetuin-A level was not investigated. The previous drawbacks should be discussed in the upcoming studies.

## Conclusion

Fetuin-A could be a reliable marker diagnostic biomarker for NAFLD. In addition, the marker was connected to the severity of the disease because it demonstrated a significant correlation with the ultrasound grading and the FibroScan using the controlled attenuated parameter.

## Data Availability

All relevant data are included in this published article.

## Abbreviations

ALT: Alanine Aminotransferase
AST: Aspartate Aminotransferase
ANOVA: Analysis of Variance
AUC: Area Under Curve
BMI: Body Mass Index
CAP scan: Controlled Attenuated Parameter scan
CIMT: Carotid Intima-Media Thickness
CK18: Cytokeratin-18
CT: Computed tomography
ELISA: Enzyme-Linked Immunosorbent Assay
F: Fibrosis
FBS: Fasting Blood Sugar
FGF-21: Fibroblast Growth Factor-21
GGT: Gamma-Glutamyl Transpeptidase
HDL: High-Density Lipoprotein
IFG: Impaired fasting glycaemia
IQR: Interquartile Range
LDL: Low-Density Lipoprotein
LSM: Liver Stiffness Measurement
MetS: Metabolic Syndrome
MRI: Magnetic Resonance Imaging
NAFLD: Non-Alcoholic Fatty Liver Disease
NFS: NAFLD fibrosis score
ROC Curve: Receiver Operator Characteristic Curve
S: Steatosis
SD: Standard Deviation
SPSS: Statistical Package for the Social Sciences
T2D: Type 2 diabetes
TG: Triglycerides

## References

[1] Dvorak K, Stritesky J, Petrtyl J, Vitek L, Sroubkova R, Lenicek M, et al. Use of non-invasive parameters of non-alcoholic steatohepatitis and liver fibrosis in daily practice-an exploratory case-control study. PLoS ONE. 2014;9(10):e111551. 25350286 10.1371/journal.pone.0111551 PMC4211730

[2] Rinella ME, Lazarus JV, Ratziu V, Francque SM, Sanyal AJ, Kanwal F, Romero D, Abdelmalek MF, Anstee QM, Arab JP, Arrese M. A multisociety Delphi consensus statement on new fatty liver disease nomenclature. Hepatology. 2023;78(6):1966–86. 37363821 10.1097/HEP.0000000000000520 PMC10653297

[3] Friedman SL, Neuschwander-Tetri BA, Rinella M, Sanyal AJ. Mechanisms of MASLD development and therapeutic strategies. Nat Med. 2018;24(7):908–22. 29967350 10.1038/s41591-018-0104-9 PMC6553468

[4] Deng J, Fishbein MH, Rigsby CK, Zhang G, Schoeneman SE, Donaldson JS. Quantitative MRI for hepatic fat fraction and T2* measurement in pediatric patients with non-alcoholic fatty liver disease. Pediatr Radiol. 2014;44:1379–87. 24840769 10.1007/s00247-014-3024-y

[5] Zhou Y, Nie M, Zhou H, Mao F, Zhao L, Ding J, Jing X. Head-to-head comparison of three different US-based quantitative parameters for hepatic steatosis assessment: a prospective study. Abdom Radiol. 2024 May;13:1–0. 10.1007/s00261-024-04347-z 38740581

[6] Icer MA, Yıldıran H. Effects of fetuin-A with diverse functions and multiple mechanisms on human health. Clin Biochem. 2021;88:1–0. 33245873 10.1016/j.clinbiochem.2020.11.004

[7] Chekol Abebe E, Tilahun Muche Z, Behaile T, Mariam A, Mengie Ayele T, Mekonnen Agidew M, Teshome Azezew M, Abebe Zewde E, Dejenie A, T. and, Mengstie A, M. The structure, biosynthesis, and biological roles of fetuin-A: a review. Front cell Dev Biology. 2022;10:945287. 10.3389/fcell.2022.945287 PMC9340150 35923855

[8] Liu S, Xiao J, Zhao Z, Wang M, Wang Y, Xin Y. Systematic review and meta-analysis of circulating fetuin-A levels in nonalcoholic fatty liver disease. J Clin Translational Hepatol. 2021;9(1):3. 10.14218/JCTH.2020.00081 PMC7868693 33604250

[9] Mathew G, Agha R. STROCSS 2021: strengthening the reporting of cohort, cross-sectional and case-control studies in surgery. IJS Short Rep. 2021;6(4):e35. 10.1097/SR9.0000000000000035 34774726

[10] Tan KC. Appropriate body-mass index for Asian populations and its implications for policy and intervention strategies. Lancet. 2004;363(9403):157–63. 14726171 10.1016/S0140-6736(03)15268-3

[11] Angulo P, Hui JM, Marchesini G, Bugianesi E, George J, Farrell GC, et al. The MASLD fibrosis score: a noninvasive system that identifies liver fibrosis in patients with NAFLD. Hepatology. 2007;45(4):846–54. 17393509 10.1002/hep.21496

[12] Saadeh S, Younossi ZM, Remer EM, Gramlich T, Ong JP, Hurley M, et al. The utility of radiological imaging in nonalcoholic fatty liver disease. Gastroenterology. 2002;123(3):745–50. 12198701 10.1053/gast.2002.35354

[13] Sasso M, Tengher-Barna I, Ziol M, Miette V, Fournier C, Sandrin L, et al. Novel controlled attenuation parameter for noninvasive assessment of steatosis using Fibroscan^®^: validation in chronic hepatitis C. J Viral Hepatitis. 2012;19(4):244–53. 10.1111/j.1365-2893.2011.01534.x 22404722

[14] Wong MC, Huang JL, George J, Huang J, Leung C, Eslam M, et al. The changing epidemiology of liver diseases in the Asia–Pacific region. Nat Reviews Gastroenterol Hepatol. 2019;16(1):57–73. 10.1038/s41575-018-0055-0 30158570

[15] Younossi ZM. The epidemiology of nonalcoholic steatohepatitis. Clin Liver Disease. 2018;11(4):92–4. 10.1002/cld.710 PMC6385947 30992797

[16] Amoroso M, Augustin S, Moosmang S, Gashaw I. Non-invasive biomarkers prognostic of decompensation events in NASH cirrhosis: a systematic literature review. J Mol Med 2024 May 16:1–8. 10.1007/s00109-024-02448-2 PMC11213726 38753041

[17] Patel K, Asrani SK, Fiel MI, Levine D, Leung DH, Duarte-Rojo A, Dranoff JA, Nayfeh T, Hasan B, Taddei TH, Alsawaf Y. Accuracy of blood-based biomarkers for staging liver fibrosis in chronic liver disease: A systematic review supporting the AASLD Practice Guideline. Hepatology. 2024 Mar 15:10–97. 10.1097/HEP.0000000000000842 38489517

[18] Mori K, Emoto M, Inaba M. Fetuin-A: a multifunctional protein. Recent Pat Endocr Metab Immune Drug Discovery. 2011;5(2):124–46. 10.2174/187221411799015372 22074587

[19] Trepanowski JF, Mey J, Varady KA. Fetuin-A: a novel link between obesity and related complications. Int J Obes. 2015;39(5):734–41. 10.1038/ijo.2014.203 25468829

[20] Ou HY, Yang YC, Wu HT, Wu JS, Lu FH, Chang CJ. Serum fetuin-A concentrations are elevated in subjects with impaired glucose tolerance and newly diagnosed type 2 diabetes. Clin Endocrinol. 2011;75(4):450–5. 10.1111/j.1365-2265.2011.04070.x 21521338

[21] Haukeland JW, Dahl TB, Yndestad A, Gladhaug IP, Løberg EM, Haaland T, et al. Fetuin-A in nonalcoholic fatty liver disease: in vivo and in vitro studies. Eur J Endocrinol. 2012;166(3):503–10. 22170794 10.1530/EJE-11-0864

[22] Peter A, Kovarova M, Staiger H, Machann J, Schick F, Königsrainer A, et al. The hepatokines fetuin-A and fetuin-B are upregulated in the state of hepatic steatosis and may differently impact on glucose homeostasis in humans. Am J Physiology-Endocrinology Metabolism. 2018;314(3):E266–73. 10.1152/ajpendo.00262.2017 29138227

[23] Godoy-Matos AF, Silva Júnior WS, Valerio CM. MASLD as a continuum: from obesity to metabolic syndrome and diabetes. Diabetol Metab Syndr. 2020;12:1–20. 32684985 10.1186/s13098-020-00570-y PMC7359287

[24] Ramadori P, Klag T, Malek NP, Heikenwalder M. Platelets in chronic liver disease, from bench to bedside. JHEP Rep. 2019;1(6):448–59. 32039397 10.1016/j.jhepr.2019.10.001 PMC7005648

[25] Nah EH, Cho S, Park H, Noh D, Kwon E, Cho HI. Subclinical steatohepatitis and advanced liver fibrosis in health examinees with nonalcoholic fatty liver disease (NAFLD) in 10 South Korean cities: a retrospective cross-sectional study. PLoS ONE. 2021;16(11):e0260477. 34818372 10.1371/journal.pone.0260477 PMC8612540

[26] Choe EK, Kang HY. The association between platelet-related parameters and nonalcoholic fatty liver disease in a metabolically healthy nonobese population. Sci Rep. 2024;14(1):6118. 38480828 10.1038/s41598-024-56796-7 PMC10937929

[27] Katsiki N, Mikhailidis DP, Mantzoros CS. Non-alcoholic fatty liver disease and dyslipidemia: an update. Metabolism. 2016;65(8):1109–23. 27237577 10.1016/j.metabol.2016.05.003

[28] Souza MR, Diniz MD, Medeiros-Filho JE, Araújo MS. Metabolic syndrome and risk factors for non-alcoholic fatty liver disease. Arq Gastroenterol. 2012;49:89–96. 22481692 10.1590/S0004-28032012000100015

[29] Lim S, Park YM, Sakuma I, Koh KK. How to control residual cardiovascular risk despite statin treatment: focusing on HDL–cholesterol. Int J Cardiol. 2013;166(1):8–14. 22503572 10.1016/j.ijcard.2012.03.127

[30] Kikkawa K, Nakajima K, Shimomura Y, Tokita Y, Machida T, Sumino H, et al. Small dense LDL cholesterol measured by homogeneous assay in Japanese healthy controls, metabolic syndrome and diabetes patients with or without a fatty liver. Clin Chim Acta. 2015;438:70–9. 25050800 10.1016/j.cca.2014.07.017

[31] Cao W, Zhao C, Shen C, Wang Y. Cytokeratin 18, alanine aminotransferase, platelets and triglycerides predict the presence of nonalcoholic steatohepatitis. PLoS ONE. 2013;8(12):e82092. 24324749 10.1371/journal.pone.0082092 PMC3853116

[32] Charatcharoenwitthaya P, Lindor KD, Angulo P. The spontaneous course of liver enzymes and its correlation in nonalcoholic fatty liver disease. Dig Dis Sci. 2012;57:1925–31. 22373863 10.1007/s10620-012-2098-3

[33] Rodríguez G, Gallego S, Breidenassel C, Moreno LA, Gottrand F. Is liver transaminases assessment an appropriate tool for the screening of non-alcoholic fatty liver disease in at risk obese children and adolescents? Nutr Hosp. 2010;25(5):712–7. 21336425

[34] Patton HM, Sirlin C, Behling C, Middleton M, Schwimmer JB, Lavine JE. Pediatric nonalcoholic fatty liver disease: a critical appraisal of current data and implications for future research. J Pediatr Gastroenterol Nutr. 2006;43(4):413–27. 17033514 10.1097/01.mpg.0000239995.58388.56

[35] Sardana O, Goyal R, Bedi O. Molecular and pathobiological involvement of fetuin-A in the pathogenesis of NAFLD. Inflammopharmacology. 2021;29(4):1061–74. 34185201 10.1007/s10787-021-00837-4

[36] Yilmaz Y, Yonal O, Kurt R, Ari F, Oral AY, Celikel CA, et al. Serum fetuin A/α 2HS-glycoprotein levels in patients with non-alcoholic fatty liver disease: relation with liver fibrosis. Ann Clin Biochem. 2010;47(6):549–53. 20926473 10.1258/acb.2010.010169

[37] Komsa-Penkova RS, Golemanov GM, Radionova ZV, Tonchev PT, Iliev SD, Penkov VV. Fetuin-A–alpha2-heremans-schmid glycoprotein: from structure to a novel marker of chronic diseases part 1. Fetuin-A as a calcium chaperone and inflammatory marker. J Biomedical Clin Res. 2017;10(2):90–7. 10.1515/jbcr-2017-0015

[38] Sato M, Kamada Y, Takeda Y, Kida S, Ohara Y, Fujii H, et al. Fetuin-A negatively correlates with liver and vascular fibrosis in nonalcoholic fatty liver disease subjects. Liver Int. 2015;35(3):925–35. 25627311 10.1111/liv.12478

[39] Peng K, Mo Z, Tian G. Serum lipid abnormalities and nonalcoholic fatty liver disease in adult males. Am J Med Sci. 2017;353(3):236–41. 28262209 10.1016/j.amjms.2017.01.002

[40] Lee GH, Peng C, Park SA, Hoang TH, Lee HY, Kim J, et al. Citrus peel extract ameliorates high-fat diet-induced MASLD via activation of AMPK signaling. Nutrients. 2020;12(3):673. 32121602 10.3390/nu12030673 PMC7146518

[41] Pan X, Kaminga AC, Chen J, Luo M, Luo J. Fetuin-A and Fetuin-B in non-alcoholic fatty liver disease: a meta-analysis and meta-regression. Int J Environ Res Public Health. 2020;17(8):2735. 32326594 10.3390/ijerph17082735 PMC7215562

[42] Kahraman A, Sowa JP, Schlattjan M, Sydor S, Pronadl M, Wree A, et al. Fetuin-A mRNA expression is elevated in NASH compared with NAFL patients. Clin Sci. 2013;125(8):391–400. 10.1042/CS20120542 23627434

[43] Filardi T, Panimolle F, Tiberti C, Crescioli C, Lenzi A, Pallotta N, Morano S. Circulating levels of fetuin-A are associated with moderate–severe hepatic steatosis in young adults. J Endocrinol Investig. 2021;44:105–10. 32350824 10.1007/s40618-020-01274-w

[44] Jung TW, Ahn SH, Shin JW, Kim HC, Park ES, Abd El-Aty AM, et al. Protectin DX ameliorates palmitate‐induced hepatic insulin resistance through AMPK/SIRT 1‐mediated modulation of fetuin‐A and SeP expression. Clin Exp Pharmacol Physiol. 2019;46(10):898–909. 31246318 10.1111/1440-1681.13131

[45] Şiraz ÜG, Doğan M, Hatipoğlu N, Muhtaroğlu S, Kurtoğlu S. Can fetuin-A be a marker for insulin resistance and poor glycemic control in children with type 1 diabetes mellitus? J Clin Res Pediatr Endocrinol. 2017;9(4):293. 28529199 10.4274/jcrpe.4532 PMC5785634

[46] Awadein MA, Makholof MA, Saleh SA, Salama MM, Hussein RM, El-Ansary AR. Assessment of serum Fetuin-A level in patients with MASLD and Chronic Hepatitis C. Egypt J Hosp Med. 2022;89(2):6282–8. 10.21608/ejhm.2022.268968

[47] Etienne Q, Lebrun V, Komuta M, Navez B, Thissen JP, Leclercq IA, Lanthier N. Fetuin-A in activated liver macrophages is a key feature of non-alcoholic steatohepatitis. Metabolites. 2022;12(7):625. 35888749 10.3390/metabo12070625 PMC9319870

[48] Lee KY, Lee W, Jung SH, Park J, Sim H, Choi YJ, et al. Hepatic upregulation of fetuin-A mediates acetaminophen-induced liver injury through activation of TLR4 in mice. Biochem Pharmacol. 2019;166:46–55. 31077645 10.1016/j.bcp.2019.05.011

[49] Schwärzler J, Grabherr F, Grander C, Adolph TE, Tilg H. The pathophysiology of MASLD: an immunometabolic perspective. Expert Rev Clin Immunol. 2024;20(4):375–86. 38149354 10.1080/1744666X.2023.2294046

[50] Filardi T, Panimolle F, Tiberti C, Crescioli C, Lenzi A, Pallotta N, et al. Circulating levels of fetuin-A are associated with moderate–severe hepatic steatosis in young adults. J Endocrinol Investig. 2021;44:105–10. 32350824 10.1007/s40618-020-01274-w

[51] Xu H, Uysal KT, Becherer JD, Arner P, Hotamisligil GS. Altered tumor necrosis factor-α (TNF-α) processing in adipocytes and increased expression of transmembrane TNF-α in obesity. Diabetes. 2002;51(6):1876–83. 12031976 10.2337/diabetes.51.6.1876

[52] Gonzalez-Gil AM, Elizondo-Montemayor L. The role of exercise in the interplay between myokines, hepatokines, osteokines, adipokines, and modulation of inflammation for energy substrate redistribution and fat mass loss: a review. Nutrients. 2020;12(6):1899. 32604889 10.3390/nu12061899 PMC7353393

